# Becoming clinical supervisors: identity learnings from a registrar faculty development program

**DOI:** 10.1007/s40037-020-00642-9

**Published:** 2020-12-28

**Authors:** Christy Noble, Jessica Young, Ellen Hourn, Dale Sheehan

**Affiliations:** 1 Faculty of Medicine and School of Pharmacy, The University of Queensland, Herston, Australia; 2 Gold Coast Health, Southport, Australia; 3 School of Medicine, Griffith University, Southport, Australia; 4 The University of Canterbury, Canterbury, New Zealand

**Keywords:** Professional identity formation, Faculty development, Clinical supervision, Educator identity formation

## Abstract

This article shares our experiences and surprises as we developed, implemented and evaluated a 12-week faculty development program for registrars as clinical supervisors over three cohorts. The program has consistently been rated highly by participants. Yet, following a comprehensive curriculum review, we were surprised that our goal of encouraging identity development in clinical supervisors seemed to be unmet. Whilst our evaluation suggests that the program made important contributions to the registrars’ knowledge, application and readiness as clinical supervisors, challenges linked to developing a supervisor identity and managing the dual identity of supervisor and clinician remain. In this article we describe our program and argue for the importance of designing faculty development programs to support professional identity formation. We present the findings from our program evaluation and discuss the surprising outcomes and ongoing challenges of developing a cohesive clinical educator identity. Informed by recent evidence and workplace learning theory we critically appraise our program, explain the mechanisms for the unintended outcomes and offer suggestions for improving curricular and pedagogic practices of embedded faculty development programs. A key recommendation is to not only consider identity formation of clinical supervisors from an individualist perspective but also from a social perspective.

**Supplementary Information:**

The online version of this article (10.1007/s40037-020-00642-9) contains supplementary material, which is available to authorized users.

## The story

Supervision is a core component of the role of hospital-based clinicians [1], yet it is acknowledged that clinicians are not always prepared to take on this role [2]. The supervisory role is complex and multifaceted [1, 3], in that it incorporates many aspects such as clinical, educational, personal and interpersonal supervision (i.e. how the supervisee interacts with others) [4]. As practice-based medical educators we identified that our registrars were experiencing challenges in understanding and enacting their role as clinical supervisors. To support the registrars in this role, we turned to the literature to inform our faculty development program. In this article we share our experiences of designing a faculty development program for registrars, its implementation and evaluation findings. We also share some surprising outcomes and relate these to current literature on identity formation.

## Program design

We were motivated to develop a comprehensive faculty development program because effective clinical supervision of junior doctors improves patient and learning outcomes [5]. Whilst acknowledging that registrars’ knowledge of educational principles and teaching strategies is highly variable [4], we sought to address the challenges of translating the new learning into practice [6]. Moreover, being mindful that the most overlooked component when designing such programs is identity formation [7], we sought to develop a program that would contribute to forming registrars’ identity. Cruess et al. define clinician identity as, “consistently demonstrates the attitudes, values, and behaviours expected of one who has come to ‘think, act, and feel like a physician’” (p. 181) [8]. Thus, a key goal for our program was to develop registrars who consistently thought, acted and felt like supervisors in complex clinical environments.

We saw strong parallels with Billett’s framing of learner *readiness* in workplace learning and identity formation, in that readiness is defined as an individual’s ability to learn from what they know (conceptual readiness), can do (procedural readiness) and value (dispositional readiness) [9]. Together these capacities of readiness mediate how the registrars make sense of their experiences both in the classroom and the workplace (e.g. as they enact their learnings about clinical supervision into practice). Another key consideration informing our program design was that readiness capacities are interdependent, in that the way we do things is determined by all three capacities [9]. For example, a registrar might understand the principles of effective feedback (conceptual) and value learner-centred feedback processes (dispositional) yet rarely enact feedback practices within the team (procedural) and, therefore, lack feedback readiness.

Given this synergy between readiness and identity formation, we aimed to design a program that developed registrars’ readiness as clinical supervisors. Our assumption was that interdependent readiness is an indicator of and contributes to identity development. Thus, we aimed to ensure that all pedagogical strategies promoted the development of each readiness capacity and we designed activities that promoted interdependent readiness, i.e. conceptual combined with procedural and dispositional readiness.

## Implementation

We selected 10 to 20 self-nominated registrars—working in either emergency or medicine subspecialties—per year for 3 years to engage in the program. The blended program had a practical focus while introducing core medical education concepts, theory and topics common to other similar programs [3]. Six modules, focusing on key supervisory roles and tasks, were delivered over 12 weeks. The topics were:

1. Orientation and setting expectations for junior doctors
2. How to enhance junior doctor learning in the clinical setting
3. Engaging in learner-centred feedback
4. Conducting effective work-based assessments
5. Delivering an excellent presentation
6. Professional identity formation and medical education

Based on our understandings of readiness and identity, each module had a common design framework to ensure the pedagogical activities developed all aspects of readiness. This framework is outlined in Table 1 of the Electronic Supplementary Material. The module materials were available online via the hospital’s learning management system. Pre-readings and face-to-face workshops—fortnightly, for 90 min—were intended to develop participants’ conceptual knowledge. Before and after the workshops, registrars were invited to engage in workplace-based learning activities (e.g. assess a trainee using a work-based assessment tool) which focused on developing their procedural readiness for teaching and supervision. Throughout the program, discussions and guided reflections with peers and medical education experts around learning experiences were conducted to support dispositional readiness. We purposefully integrated pedagogical activities into the modules where evidence suggests they make important contributions to identity formation, including drawing comics [10], facilitated discussions and storytelling [11] and reflective writing [12, 13] with the final module—Professional Identity Formation and Medical Education—aiming to integrate these activities and concepts.

## Evaluation process

Our qualitative evaluation focused on ‘participants learning’, i. e., whether registrars’ learner readiness had improved throughout the program [14]. As analysis of personal reflections is considered a valid way to assess participant learnings [14], registrars’ reflections from each module and the 6‑month follow-up reflection survey informed our evaluation. The module reflections, as illustrated in Table 1 of the Electronic Supplementary Material, asked registrars to write about their experiences, how they implemented their learnings and what they learnt as a result. Similar questions were used in the 6‑month follow-up reflection survey as well as questions related to longitudinal changes in teaching practices and ongoing challenges related to integrating learnings into practice. These data (i.e. responses to reflections and follow-up survey) were collated, coded and analysed thematically using learner readiness (i.e. conceptual, procedural and dispositional) [9] as a theoretical framework. The initial data coding and analysis was performed by CN and EH who discussed the themes with the rest of the team. Our local ethics committee confirmed that this was a quality activity (HREC/16/QGC/117).

## Outcomes

We found that all registrars’ readiness improved. In terms of conceptual readiness, they described the importance of assessing learners’ needs and responding to teaching opportunities in practice. Participants gave clear descriptions on how to structure bedside teaching sessions and engage in effective feedback practices.

In terms of procedural readiness development, the registrars were enacting their role as supervisors by generating practical strategies to orientate the juniors, such as using checklists. They described refining their communication skills when teaching to ensure learner engagement and to avoid cognitive overload. When assessing their juniors, registrars described selecting appropriate tools.

**Fig. 1:**
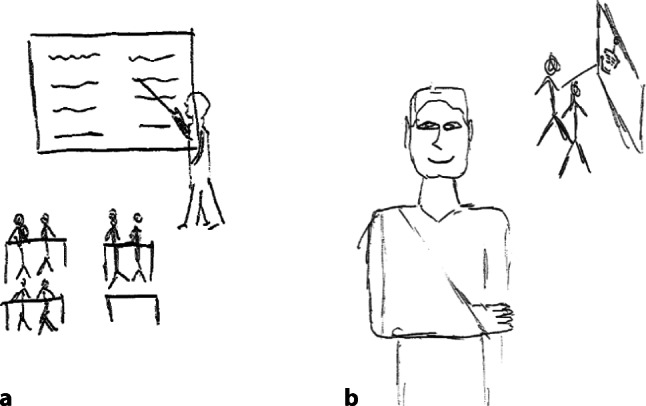
Changing conceptualisation of supervisor role. **a** Before, **b** After

For dispositional readiness, the registrars’ reflections demonstrated they were adopting dispositions aligned to effective supervision. For example, they noted being effortful in improving trainee learning opportunities and promoting safe learning environments. They described adopting an inclusive approach to teaching and learning, such as taking new interns for coffee so they felt part of the team and striving towards learner-centred approaches to pedagogical practices. Their comics demonstrated new ways of understanding their supervising role, for example from a didactic and formal interaction towards a learner-centred, opportunistic approach. An example of a registrar’s comic is presented in Fig. 1.

Overall, these findings suggested that our program made important contributions to developing registrars as clinical supervisor. But some findings caught us by surprise.

## Surprising outcomes

Our surprising outcome was that registrars were struggling to enact their teacher identities in practice. Evidence of this challenge was interwoven across all module reflections and the follow-up survey and included: 1) balancing practice with teaching; 2) reconciling their teacher identity with co-working and 3) lacking agency to implement improvements.

### Balancing practice with teaching

Whilst the registrars were episodically translating their learnings into practice, they consistently reflected that a key challenge was sustaining the integration into practice in busy clinical settings. The following quote illustrates a typical response:

Given how busy the department has been lately it is often hard to find the time to use the skills (Registrar 3).

### Reconciling teacher identity with co-working

The registrars noted that it was challenging to be both a clinical team member and a supervisor. Some tensions included not being able to fully engage in feedback processes and not wanting to upset team members with feedback:

… [I] did not provide enough constructive criticism, likely due to bias (we are friends and I like her) (Registrar 5).

Another reconciliation challenge described was that the registrars were still developing as clinicians whilst needing to support the juniors. Moreover, they conceptually understood the importance of supporting trainees, yet found it challenging to enact this role. For example:

When I reflect on the first term of this year, I must admit that my involvement in supporting the induction of interns was very limited. This was partially due to the fact that I also needed a lot of support at the beginning of this year to familiarise with unit organisation and with expectations of my new role as an advanced trainee (Registrar 1).

### Lacking agency to implement improvements

Whilst the registrars were recognising opportunities to improve teaching, they consistently noted a lack of agency to implement meaningful changes to improve juniors’ learning. For example, one registrar expressed concerns about assessment practices being tokenistic but did not articulate strategies for improving:

Often, however, seems to be a box-ticking exercise when time pressures and stress play a role … seems like an arbitrary exercise with little benefit when utilised on the ward (Registrar 1).

Finally, for each cohort the attrition rate was approximately 30% and compliance with completing the reflections fell off over time. These behaviours were attributed to needing to prioritise clinical responsibilities. Having conducted our curriculum review, this observed attrition likely further confirms that integration of teacher identity is challenging especially when clinician identity is being privileged.

## Lessons learned

Given this surprising outcome, we realised that we needed to reconsider our understanding of teacher identity formation. Our key lessons learned, informed by a recent literature review [15], provided important insights into the nature of clinical teacher professional identity formation. We were reminded that clinicians developing teacher identities are also juggling this with their clinical and sometimes research identities [15]. Moreover, this process occurs in hierarchical social settings where teaching and supervision may not be the highest priority. To summarise, findings from the literature review suggest that:

the scholarship of identity can be conceptualized as an epistemic continuum between, at one end, individualist (positivist) perspectives that situate identity development within the person and, at the other, social relational (relativist) standpoints that conceptualize identity as constructed through social interaction in cultural contexts [15].

Our standpoint had been primarily individualistic and with less consideration given to the social relational perspective, particularly the idea that supervisor identity is negotiated in social contexts.

To address our surprising outcome, we are now reviewing new ways to support identity development within both social and cultural contexts whilst retaining individualistic strategies. Based on Cantillon et al.’s [15] key themes related to supporting teacher identity formation from a social relational perspective and cultural context and through email discussions with P. Cantillon in August 2019, some of our practical strategies, are described below.

### Identity as contingent (“How teacher identity is contingent on social acknowledgement and support” p. 1613) [15]

Implementing an awards program within our organisation to recognise and celebrate teaching excellence. Carefully considering how routine teaching practices, e. g., consultants lead orientations for new junior doctors, can include registrars.

### Identity as communicated (“How identity is communicated between teachers and clinical peers” p. 1613) [15]

Encouraging registrars to practise teaching conversations, e.g. sharing their teaching experiences and ideas, within their clinical teams. Also, extending these conversations so that registrars seek guidance and feedback from local expert clinical teachers as means to further generate dialogue and improve their supervisory practice [16].

### Identity as negotiated (“How teacher identity is co-constructed between individuals and workplace contexts” p. 1613) [15]

Implementing department-wide faculty development programs including all doctors on particular pedagogic practices, such as feedback and bedside teaching. Within our emergency department we have developed and implemented a development program on feedback practices where: 1) the interns are engaging in a feedback literacy program to encourage them to seek feedback [17, 18] whilst, 2) the supervisors and registrars are engaging in faculty program encouraging them to seek feedback on their feedback.

### Identity as organisationally informed (“How identity is shaped by organizational culture” p. 1613) [15]

We are planning faculty development presentations as part of Grand Rounds and are liaising with the national health professional education organisations to raise awareness of this teaching community and mentor support program for registrars.

The practical strategies (described above) can be categorised as micro or macro level changes. To support clinical teacher identity formation, our key lesson is to implement changes at micro and macro levels [16], that is: 1) what individuals (e.g. registrars) can do; and 2) what organisations can do. For individuals, this likely requires development of capabilities to: a) address conflicting priorities (i.e. balancing service and teaching) and b) strategies to enhance their agency to implement improvements. Firstly, overcoming challenges related to conflicting priorities could be addressed by aiding new clinical teachers’ understanding that much learning occurs through practice and is often invisible [19, 20]. Innovative methodologies, such as video reflexive ethnography (VRE) rather than traditional faculty development programs, could illuminate these learning opportunities and support the generation of strategies to facilitate learning through practice [21, 22], thereby supporting registrars to become more aware of themselves as expert guides [23].

Secondly, enhancing registrars’ agency, as becoming supervisors, will require deliberate reflection on what the organisational culture (e.g. hierarchy) is implicitly telling them about clinical supervision. Then working with them to identify ‘teaching workarounds’ whilst still delivering institutional priorities, such as clinical care. Conceptually, this means generating learning curriculum [23] which journeys registrars from *having* the appropriate disposition (e.g. developing the motivation to identify ‘teaching work arounds’) to *enacting* the appropriate disposition (e.g. implementing the ‘teaching work arounds’). Thereby, supporting identity formation through an iterative process of working with individuals as they engage with and respond to social and cultural contexts.

From an *organisational* perspective, teams or institutions need to become more reflexive and aware of the cultural effects on teacher identity formation. For example, giving new registrars a supportive platform for communicating about teaching experiences can illuminate their fresh perspectives and offer insights for fostering tailored learning experiences. As noted above, using strategies such as VRE or brief ethnographic observations, could illuminate organisational culture norms and the effects these have on clinical supervision practices. By illuminating these norms, practice-based strategies to foster trainees’ development as clinical supervisors can be generated. Programs like ours can highlight the need for feedback, beyond the immediate clinical team, to inform and improve the broader clinical education system. Overall, we believe we can do more. We are aiming to not just embed the program within our social and cultural context but also foster the formation of a supervisor community of practice within the service to better support and develop the identity of supervisors.

## Moral of the story

Through our evaluation and reflection, we recognise that our initial belief that readiness promotes identity formation generated an individualist approach to supervisor identity formation. We had not explored how social and cultural factors contributed to, and could enhance or hamper, supervisor identity formation. Indeed, the clues were apparent with registrars saying it is challenging to integrate clinical teaching in busy workdays. Thus, we are very mindful that it is important to not ignore the clues or niggles and to consider social and cultural factors. Moreover, if these niggles emerge then they require further exploration and careful consideration about the realities of practice. Finally, there is great value in engaging expert insights (in our case, a contemporary literature review [15] and emails with the author) and exploring their insights to identify practical solutions. In short, model what you expect of your supervisors-in-training and seek expert guidance.

## Supplementary Information

Table 1: Structure of modules and contributions to readiness
