# Supplementary material for: Becoming clinical supervisors: identity learnings from a registrar faculty development program
Source: Perspect Med Educ. 2020 Dec 28;10(2):125–9. doi: 10.1007/s40037-020-00642-9 (PMC7952496; doi:10.1007/s40037-020-00642-9)
Supplement: Supplementary file 1 — Table 1: Structure of modules and contributions to readiness [file 40037_2020_642_MOESM1_ESM.docx]

Table 1: Structure of modules and contributions to readiness

| **Module Phase** | **Pedagogical Activity** | **Purpose of pedagogical activity** | **Example from Module** | **Likely contributions to readiness** |
| --- | --- | --- | --- | --- |
| 1 | Pre-reading based on best medical education evidence | To introduce registrars to the conceptual knowledge related to the module topic. | **Reading:**  Mulroy S, et al. (2007). "What do junior doctors want in start-of-term orientation?" Medical Journal of Australia **186**(7): S37-S39.  This article provided an overview of junior doctors' expectations of their orientation to a new term.  Read the article prior to coming to the face to face session and generate one (1) critical question to be presented at the workshop | Conceptual |
| 2 | Pre-workshop activity (workplace focussed) | To promote engagement with the module topic and to assist with relating the learnings from Phase 1 to their personal workplace experiences. | **Activity:** Ask the junior doctors or medical students in your team about the following:   - How were they oriented to the team? - What was the most useful aspect? What important aspect was missed? - Explore whether they were made to feel part of the team and when this occurred i.e. what precipitated that sense of belonging?   You will be asked to present these findings in Session 1. | Conceptual and Procedural |
| 3 | Face to face workshop | To create opportunities to discuss ideas and learnings from Phase 1 and 2; and to augment learning through discussion with and role modelling of medical education experts facilitating the workshop. | Facilitated discussion and activities to:   - Introduce concepts related to the theory and practice of clinical supervision - Describe purpose of clinical supervision - Be able to agree expectations and orientate junior doctors to a new term - Outline the purpose and structure of a ‘good orientation’ | Conceptual, Procedural and Dispositional |
| 4 | Post-workshop activity (workplace focussed) and Reflection | To provide learners with an opportunity to integrate learnings to their clinical context and to consolidate these experiences. | Post-workshop activity:   - Either orientate your new junior doctor or ask them about their orientation (questionnaire provide and was based on Thomson et al, 2014) - Draft some recommendations for how the orientation might be improved in your unit/team.   Guided reflection:  Please list three (3) key ideas you took away from the orientation session that you will now utilise in your practice. | Conceptual, Procedural and Dispositional |
